# Rapid diagnostic tests for malaria

**DOI:** 10.2471/BLT.14.151167

**Published:** 2015-09-25

**Authors:** Theodoor Visser, Jennifer Daily, Nora Hotte, Caitlin Dolkart, Jane Cunningham, Prashant Yadav

**Affiliations:** a Clinton Health Access Initiative, Inc., 383 Dorchester Avenue, Suite 400, Boston, MA 02127, United States of America (USA).; b Consultant to UNITAID, Denver, USA.; c Global Malaria Programme, World Health Organization, Geneva, Switzerland.; d William Davidson Institute, University of Michigan, Ann Arbor, USA.

## Abstract

Maintaining quality, competitiveness and innovation in global health technology is a constant challenge for manufacturers, while affordability, access and equity are challenges for governments and international agencies. In this paper we discuss these issues with reference to rapid diagnostic tests for malaria. Strategies to control and eliminate malaria depend on early and accurate diagnosis. Rapid diagnostic tests for malaria require little training and equipment and can be performed by non-specialists in remote settings. Use of these tests has expanded significantly over the last few years, following recommendations to test all suspected malaria cases before treatment and the implementation of an evaluation programme to assess the performance of the malaria rapid diagnostic tests. Despite these gains, challenges exist that, if not addressed, could jeopardize the progress made to date. We discuss recent developments in rapid diagnostic tests for malaria, highlight some of the challenges and provide suggestions to address them.

## Résumé

Le maintien de la qualité, de la compétitivité et de l'innovation dans les technologies de la santé au niveau mondial représente un défi constant pour les fabricants, tandis que l'accessibilité économique, l'accès et l'équité constituent un défi pour les gouvernements et les organismes internationaux. Nous abordons ces questions dans le présent rapport en ce qui concerne les tests de diagnostic rapide du paludisme. Les stratégies visant à enrayer et à éradiquer le paludisme reposent sur un diagnostic précoce et précis. Les tests de diagnostic rapide du paludisme nécessitent peu de formation et de matériel et peuvent être réalisés par des non-spécialistes dans des endroits isolés. L'utilisation de ces tests s'est considérablement répandue au cours des dernières années suite aux recommandations préconisant d'examiner tous les cas présumés de paludisme avant la mise en route d'un traitement et de mettre en œuvre un programme d'évaluation pour mesurer les performances des tests de diagnostic rapide du paludisme. Il demeure, malgré ces avancées, des défis, qui, s'ils ne sont pas surmontés, risquent de compromettre les progrès accomplis jusqu'à présent. Le présent rapport fait état des améliorations récemment apportées aux tests de diagnostic rapide du paludisme, souligne un certain nombre de défis et propose des solutions pour les surmonter.

## Resumen

Mantener la calidad, competitividad e innovación en la tecnología de salud mundial es un desafío constante para los fabricantes, mientras que la asequibilidad, el acceso y la equidad son desafíos para los gobiernos y las agencias internacionales. En este artículo se debaten estas cuestiones con relación a las pruebas de diagnóstico rápido del paludismo. Las estrategias para controlar y eliminar el paludismo dependen de un diagnóstico temprano y preciso. Las pruebas de diagnóstico rápido del paludismo requieren muy poco entrenamiento y equipo y pueden ser llevadas a cabo por no especialistas en lugares apartados. La utilización de estas pruebas se ha expandido significantemente durante los últimos años, tras las recomendaciones de hacer una prueba en todos los casos de posible paludismo antes del tratamiento y la implementación de un programa de evaluación para examinar el rendimiento de las pruebas de diagnóstico rápido del paludismo. A pesar de estos avances, todavía existen desafíos que, si no se abordan, podrían poner en peligro el progreso hecho hasta la fecha. Se debaten los recientes desarrollos de las pruebas de diagnóstico rápido del paludismo, se destacan algunos de los desafíos y se proporcionan sugerencias para tratarlos.

## ملخص

يمثل الحفاظ على مستوى الجودة، والقدرة التنافسية، والابتكار في مجال الصحة العالمية تحديًا أمام الجهات المصنعة، بينما تواجه الحكومات والوكالات الدولية تحديات أخرى تتمثل في الإنصاف، ومعقولية الأسعار، وإمكانية الحصول على الخدمات. ونناقش في هذا التقرير هذه القضايا فيما يتعلق بشأن الاختبارات السريعة لتشخيص الملاريا. تعتمد استراتيجيات السيطرة على مرض الملاريا والقضاء عليه على التشخيص المبكر والدقيق للمرض. وتتطلب الاختبارات السريعة لتشخيص الملاريا القليل من التدريب والمعدات، كما يمكن إجراؤها على يد غير المتخصصين في المناطق النائية. وقد اتسع نطاق استخدام هذه الاختبارات بصورة ملحوظة على مدى السنوات القليلة الماضية نتيجة التوصيات باختبار جميع حالات الاشتباه بالإصابة بمرض الملاريا قبل البدء في العلاج وتنفيذ برنامج للتقييم لتقدير مستوى الأداء المتعلق بالاختبارات السريعة لتشخيص الملاريا. وعلى الرغم من هذه المكاسب، ما زالت التحديات قائمة للحد الذي قد يؤدي إلى خسارة ما تم إحرازه من تقدم حتى الوقت الحالي، إن لم تتم مواجهتها. ونناقش التطورات الأخيرة المتعلقة بالاختبارات السريعة لتشخيص الملاريا، ونلقي الضوء على بعض التحديات، ونقدم بعض الاقتراحات لمواجهتها.

## 摘要

保持全球卫生技术的质量、竞争力和创新是制造商不断面临的挑战，而政府和国际机构则面临着支付能力、可近性和公平性的挑战。本文将探讨关于疟疾快速诊断测试的这些问题。控制和消除疟疾的策略取决于及早、准确的诊断。而疟疾快速诊断测试几乎不需要培训和设备，且可由非专业人员远程执行。过去几年内，随着治疗前测试所有疑似疟疾病例的建议的提出和疟疾快速测试性能评估项目的实施，这些测试的使用范围已显著扩大。尽管取得了这些成就，仍然存在一些挑战，如果不能妥善化解，它们可能危及目前为止所取得的进展。我们将探讨疟疾快速诊断的最新发展，突出其中一些挑战，并提出应对这些挑战的建议。

## Резюме

Поддержание качества, конкурентной способности и инноваций в области технологий мирового здравоохранения на высоком уровне — это задача, над решением которой постоянно работают изготовители, в то время как задачей правительств и международных организаций становится обеспечение доступности, ценовой приемлемости и равенства в вопросах лечения. В этой статье рассматриваются проблемы, связанные с быстрыми способами тестирования при постановке диагноза «малярия». Выбор стратегий контроля и искоренения малярии зависит от возможности ранней и точной постановки диагноза. Диагностические экспресс-тесты на малярию не требуют длительного обучения или специального оборудования и могут выполняться даже неспециалистами в условиях отдаленных районов. За последние несколько лет такие тесты получили широкое распространение после того, как было рекомендовано проверять все случаи подозрения на малярию до начала лечения, а также после осуществления программы оценки, направленной на определение эффективности экспресс-тестов для диагностики малярии. Несмотря на успехи, существует ряд проблем, которые могут поставить под угрозу достигнутый на сегодня прогресс, если не уделять им должного внимания. В статье рассматриваются недавние разработки в области диагностического экспресс-тестирования на малярию, освещаются некоторые из проблем, а также предлагаются способы реагирования на эти проблемы.

## Introduction

In the 1950s, the World Health Organization (WHO) launched a global malaria eradication programme based primarily on a strategy of vector control with insecticides supplemented with mass drug administration to people living in malaria-endemic countries outside sub-Saharan Africa.^1^ While nearly 40 countries succeeded in eliminating malaria in the 1950s and 1960s, many did not, and the programme eventually collapsed.^2^^,^^3^ In recent years, a renewed interest in, and commitment to, malaria elimination has emerged.^4^ WHO developed the global technical strategy that aims to eliminate malaria in at least 35 countries by 2030 in close coordination with the Roll Back Malaria Partnership.^5^

In the absence of accurate diagnostic tests, presumptive treatment or clinical diagnosis of malaria can result in the treatment of patients who do not actually have malaria,^6^^,^^7^ contributing to overtreatment, wasted resources^8^ and antimalarial medicine resistance.^9^ Since the early 1900s, microscopy has been the primary method used to diagnose malaria.^2^ In this paper, we examine the introduction of a new method to diagnose malaria – a rapid diagnostic test that uses monoclonal antibodies to detect malaria antigens in a drop of the patient’s blood. We describe recent developments that have spurred the growth in demand for rapid diagnostic tests for malaria, identify challenges that could limit further progress and make recommendations that could help mitigate these challenges.

## Rapid diagnostic tests

Quality assured microscopic examination of Giemsa-stained blood smears detecting malaria parasites is the gold standard in malaria diagnosis.^2^ However, microscopy requires trained technicians, a basic laboratory infrastructure and quality equipment and reagents. Because of these requirements, field microscopy is often performed inaccurately, especially in rural settings or in places where little malaria is found.^2^^,^^10^^,^^11^

In the 1990s, new methods to detect malaria were introduced, including molecular methods that can detect infections at very low parasite levels and antigen detection that can be used by remote health workers in areas where microscopy is impractical.^2^ Unlike microscopy or molecular methods, rapid diagnostic tests require little training and the result is available after 15 to 30 minutes. Furthermore, rapid diagnostic tests require no laboratory infrastructure, allowing them to be deployed to the most remote settings. Table 1 provides an overview of the available diagnostic methods for malaria.

**Table 1:** Malaria diagnostic technologies

Method	Description	Use	Characteristics
Antigen detection (rapid diagnostic test)	Disposable tests that detect antigens produced by malaria parasite; requires minimal training	319 million rapid diagnostic tests in 2013^a^	Rapidly growing market; size and growth attractive to suppliers; relatively low unit cost; research and development incremental, e.g. improving existing products to meet minimum standards
Microscopy	Direct visualization of parasite using microscope and stained slides; requires trained technicians	Approximately 197 million slides read and reported in 2013^a^	Mature, stable market; multiple suppliers of equipment, regents and consumables; low unit cost (especially at high volumes); research and development largely focused on developing automated systems
Nucleic acid testing	Detection of parasite ribonucleic or deoxyribonucleic acid; requires sophisticated laboratory, instruments and trained technicians	Small niche market; used for research, surveillance and as reference standard	Little standardization of methods, limited availability of commercial test kits; relatively high unit cost per test; research and development focuses on development of point-of-care devices and test kits, rather than low cost

^a ^World Malaria Report 2014. Geneva: World Health Organization; 2014.

In subsequent years, the large potential market for malaria diagnostics attracted many suppliers: by 2005 about 60 manufacturers were supplying rapid diagnostic tests.^12^ Results from field trials suggested highly variable performance but there was no system in place at a global level to independently assess and compare the performance of the tests.^13^

In 2008, WHO, the US Centers for Disease Control and Prevention and the Foundation for Innovative Diagnostics jointly implemented an evaluation programme, which coupled pre-purchase product evaluation (product testing) with a post-purchase lot verification service, to assess, review and compare the performance of malaria rapid diagnostic tests in a standardized manner. In this product testing scheme, tests provided by ISO13485-certified manufacturers are rigorously evaluated against blood samples containing *Plasmodium falciparum* or *Plasmodium vivax* and malaria-negative samples. The rapid diagnostic tests also undergo limited heat stability testing (incubated for 60 days at 35 °C and 45 °C). This challenge covers the upper limit of storage conditions recommended by rapid diagnostic test manufacturers (i.e. 35–40 °C); but is shorter than the typical product shelf-life of 18–24 months.^14^

The product evaluation programme demonstrated convincingly that some rapid diagnostic tests can consistently detect malaria at the lower limits of clinically significant disease and with very low false-positive rates.^15^ These findings were confirmed in future rounds of testing and have contributed to the broader acceptance of rapid diagnostic tests as a reliable diagnostic tool. WHO has established recommended minimum rapid diagnostic test performance criteria to inform procurement (Box 1). Major procurers – including other United Nations agencies, the Global Fund to fight AIDS, Tuberculosis and Malaria and the President’s Malaria Initiative – have adopted these criteria. The use of rapid diagnostic tests shifted to products that scored highly in the evaluation programme, a trend that continues in the public sector today.

Box 1 **World Health Organization procurement criteria for malaria rapid diagnostic tests**

Products should be selected in line with the following set of criteria, based on the results of the assessment of the World Health Organization malaria product testing programme:

1. For the detection of *Plasmodium falciparum* in all transmission settings the panel detection score against *P. falciparum* samples should be at least 75% at 200 parasites/μL.
2. For the detection of *Plasmodium vivax* in all transmission settings the panel detection score against *P. vivax* samples should be at least 75% at 200 parasites/μL.
3. There should be less than 10% false-positive test results and less than 5% invalid results.

Only products meeting performance criteria outlined above are recommended for procurement.

The results of the five evaluation rounds published so far, including 147 unique products, show that tests for *P. falciparum* that target the histidine rich protein II antigen have the highest detection scores at low parasite density. A smaller number of tests detecting *P. vivax*, targeting plasmodium lactate dehydrogenase or aldolase antigens also perform well.^15^ Most high-performing rapid diagnostic tests are also heat stable.^15^ These results are generally consistent with recent studies evaluating the performance of rapid diagnostic tests in the field.^16^^,^^17^

### Demand for rapid tests

In 2010, WHO began to recommend confirmation of all suspected malaria cases by microscopy or rapid diagnostic test before treatment^18^ and subsequently WHO and the Roll Back Malaria Partnership set ambitious targets to achieve universal access to testing for all patients suspected of having malaria.^19^ As a result, there has been a rapid increase in diagnostic testing for malaria in recent years, primarily driven by the uptake of rapid diagnostic tests in the public sector. Global sales volumes for rapid diagnostic tests rose from 48 million in 2008, to 319 million in 2013 and in many settings rapid diagnostic test use surpassed microscopy for malaria diagnosis.^20^^,^^21^ Rapid diagnostic tests are also used in private retail settings in countries where this sector plays a significant role in malaria case management.^22^^–^^24^

Rapid diagnostic tests also play an important role in detecting clinically significant malaria in settings that have reduced their malaria transmission to very low levels.^25^ There is an emerging need for tests that can detect malaria in the asymptomatic population with even lower levels of infection.^26^^,^^27^ There are uncertainties about the optimal limit of detection and trade-offs between performance, ease of use and the time taken to obtain test results.^28^ The use of rapid tests will depend on the malaria elimination strategies that countries chose to adopt, including whether and to what effect mass screening and treatment strategies are deployed.^29^ Finally, given the decline in malaria burden, most malaria tests will be negative and the current generation of rapid diagnostic tests presents health workers with the challenge of diagnosing and managing fevers that are not caused by malaria. Experience is growing with algorithmic approaches to managing these cases.^30^ New diagnostic tools for other febrile illnesses and for biomarkers that can help identify severe disease may be available in the future.^31^ In summary, we expect that demand for rapid diagnostic tests will continue to expand. Despite these gains, UNITAID’s market landscape reports^32^ as well as recent procurement and costing data analyses highlight potential challenges that, if unaddressed, jeopardize the progress made to date.

### Future challenges

A limited number of manufacturers supply the global public health market, despite a large number of eligible manufacturers. Because the process for achieving WHO prequalification status has been slow in recent years, most countries and international funders continue to use procurement criteria based on results from the WHO testing programme. While 29 manufacturers meet WHO’s recommended procurement criteria, data from the Global Fund and the President’s Malaria Initiative indicate that three manufacturers won 92% of tenders in 2013. Furthermore, since one of the three highest-volume manufacturers procures major components from one of the other two manufacturers, 92% of the public sector supply is essentially dependent on only two manufacturers.^32^ Although the two manufacturers have sufficient annual production capacity to meet current global demand, quality or capacity issues at one manufacturer could cause global supply shortages.

From 2010 to 2014, ex-factory prices for rapid malaria tests decreased by nearly 50%. Recent procurement data show that prices in some tenders are as low as 0.18 United States dollars (US$) per test.^33^ While price reductions were expected due to competition and economies of scale, the recent prices may be too low. The cost of production is estimated to be US$ 0.16 to US$ 0.23, depending on the level of automation, production volumes and allocation of indirect costs. Low prices for the public sector have caused some manufacturers to exit the market; for those that remain, the low margins may discourage research and development. Low prices could compromise quality and ultimately performance, by leading manufacturers to cut corners in their production.^34^^,^^35^

Most procurers award annual tenders to a single manufacturer. This results in shorter lead times and higher production and transport costs for manufacturers. Because manufacturers do not know if they will win or lose a tender, they are unable to accurately plan production. Given uncertain demand and low margins, most manufacturers carry little stock of raw materials and finished products. Once a tender is won, manufacturers have to rapidly procure materials at higher prices due to spot contracts and higher transport costs to ship the materials immediately. Furthermore, manufacturers experience busy months producing at capacity using multiple shifts a day to fill a tender, which in turn results in increased labour costs.

Despite WHO’s product testing programme, gaps still exist in quality control and quality assurance. With respect to product testing, manufacturers can produce (or procure) rapid diagnostic test batches exclusively for submission to the product testing programme. In addition, although nearly all lots tested pass the minimum performance thresholds in lot testing, many of the rapid diagnostic tests are not randomly sampled, since they are often sent directly by the manufacturer to the testing laboratory. In malaria endemic countries, post marketing surveillance is nearly non-existent; there are no practical tools that can be used by central reference laboratories or at point-of-care to check that rapid diagnostic tests are still performing acceptably after they are delivered. Few countries have a robust surveillance system to monitor rapid diagnostic test use in facilities and to document abnormalities and failures.^36^

A weak regulatory environment at the country level may lead to substandard rapid diagnostic tests being imported and deter manufacturers from entering the market.^36^ Lengthy registration processes, which have to be completed separately in each country, also act as barriers to market entry. In some countries, different authorities or professional associations regulate the sale and use of in vitro diagnostics, but roles may overlap and resources may be insufficient to implement the regulatory controls. If substandard rapid diagnostic tests enter the market without regulatory controls being enforced, manufacturers will need to compete against these products. In Uganda, for example, almost 20% of private pharmacies carried rapid diagnostic tests that had not been evaluated by WHO’s testing programme.^37^ Many countries have yet to determine which outlet types or providers can administer or sell rapid diagnostic tests. These decisions will affect access, especially to diagnosis by the private sector, which treats an estimated 30–50% of people with fever in countries where malaria is common.^21^

## Recommendations

To address some of the challenges described above, we propose the following recommendations. First, donors, procurement bodies and countries should increase their focus on quality. Although price will continue to be a driving force, it should not be the key factor that determines public tender selection. Since 2009, donors and major procurers have aligned rapid diagnostic test selection criteria that emphasize minimum performance requirements and lot testing. Eventually, they could also publicly agree to buy only WHO prequalified rapid diagnostic tests so as to encourage manufacturers to achieve this high standard. Recently, the major manufacturers have prequalified five rapid diagnostic tests for malaria.^38^ Procurers could hire sampling agents to randomly select rapid test products. Donors and procurers could also prioritize the adoption and roll out of quality control tools (e.g. positive controls or recombinant antigen reference panels) to support post market surveillance.

Second, the global malaria community should work together to harmonize rapid diagnostic tests with international standards and evidence to promote best practices in production, packaging and test procedures. In 2013, the Roll Back Malaria Partnership studied how rapid diagnostic tests could be harmonized to promote appropriate use and reduce operator errors. Best practices for device labelling, packaging, accessories and instructions for use were developed. Future efforts could include harmonization of test procedures, reducing the need for re-training following the introduction of new tests, but the technical and regulatory implications of such changes require careful consideration.

Third, long-term agreements with several manufacturers could contribute to low prices without putting manufacturers out of business. If manufacturers obtain volume commitments for at least one or two years, cost efficiencies can be achieved through reduced prices of materials, lower labour costs, improved production schedules and better inventory management. In pursuing these longer-term agreements, volumes should be split appropriately between eligible manufacturers.

Fourth, donors, countries and the global malaria community could work to strengthen national legal frameworks for regulation of in vitro diagnostic tests.^39^ Wherever possible, adoption of harmonized registration requirements, quality standards and regional mechanisms (e.g. regional networks of accredited laboratories to facilitate batch testing or other quality control activities) could promote best practices, avoid duplication of efforts and minimize the burden on regulators and manufacturers alike.^40^ In the short term, there is a need to provide manufacturers and importers with information on how to register rapid diagnostic tests and stipulate who may perform the test under what conditions. Given the scale up of rapid diagnostic tests for malaria and the continuing need for innovation, the stakes are particularly high. Our recommendations can help enhance quality, reduce costs, encourage innovation and increase the availability of rapid diagnostic tests.
